# Bivariate joint models for survival and change of cognitive function

**DOI:** 10.1177/09622802221146307

**Published:** 2022-12-26

**Authors:** Shengning Pan, Ardo van den Hout

**Affiliations:** Department of Statistical Science, University College, London, UK

**Keywords:** Joint model, bivariate binomial distribution, cognitive function, survival analysis, shared random-effects model

## Abstract

Changes in cognitive function over time are of interest in ageing research. A joint model is constructed to investigate. Generally, cognitive function is measured through more than one test, and the test scores are integers. The aim is to investigate two test scores and use an extension of a bivariate binomial distribution to define a new joint model. This bivariate distribution model the correlation between the two test scores. To deal with attrition due to death, the Weibull hazard model and the Gompertz hazard model are used. A shared random-effects model is constructed, and the random effects are assumed to follow a bivariate normal distribution. It is shown how to incorporate random effects that link the bivariate longitudinal model and the survival model. The joint model is applied to the English Longitudinal Study of Ageing data.

## 1 Introduction

In ageing research, data from various tests of cognitive functioning are often collected as longitudinal data, i.e. the same sample is tracked at different points in time. Generally, longitudinal data on ageing research are multi-dimensional data that contain observations for multiple phenomena obtained over multiple time periods for the same individual.^1^ For instance, the English Longitudinal Study of Ageing (ELSA) contains several cognitive tests relating to literacy, numeracy, memory and information processing.^2^ Individuals are tested every few years and the test scores are recorded; these test scores are in the form of non-negative integers. An important research topic in ageing research is the change in cognitive function over time. In our model, we present an extension of the binomial model to measure the change in non-negative cognitive test scores.

Cognitive function, also known as cognitive ability, is the individual’s ability to process information, mainly with regard to learning and problem-solving. Researchers are committed to obtaining scores that reflect the level of cognitive function of individuals through different tests.

In ageing research, it is important to investigate the changes in the individual’s cognitive function. The relationship between changes in cognitive function and the risk of death is also of interest.^3^ Researchers provide advice on whether the elderly need care by analyzing the relationship between cognitive function decline and ageing.^4^ For example, an individual whose cognitive function drops suddenly during a period might suffer from a disease such as Alzheimer’s disease that affects cognitive abilities or even threatens life, and he/she needs professional medical care. The work in Laird and Ware,^5^ Cox^6^ and Van Den Hout and Muniz-Terrera^7^ model longitudinal data and time-to-event data separately, however, if the two response processes are correlated this may lead to biased effect size estimates.^8^ Therefore, it is reasonable to use a joint model to measure both processes simultaneously. The use of joint models is not limited to the ageing research. Previous research has shown that joint modelling can improve the efficiency of statistical inference and reduce bias, and it can provide significant benefits when designing experiments.^9,10^

Research into the joint modelling of longitudinal and time-to-event data has received considerable attention over the past three decades.^3,11^ Faucett and Thomas, and Wulfsohn and Tsiatis wrote seminal articles in this field in 1996 and 1997 respectively, and after that extensions to joint models have been proposed in the literature.^12^ In the early stages of research on joint models, research on joint models has focused on single longitudinal responses and single time-event responses, i.e. univariate joint model. This approach has been widely used in clinical research^13^ and there are a number of mainstream software packages available for calculating related problems.^14–16^ However, as we mentioned above, longitudinal and time-to-event data, especially the longitudinal part, are usually multidimensional and contain observations of multiple phenomena obtained by the same person over multiple time periods. While univariate joint models allow us to determine the relationship between a survival outcome and a single longitudinal outcome, more frequently, multiple biomarkers are associated with the event of interest.^12^ Multivariate joint models allow us to consider more information simultaneously, thus enabling us to better understand the dynamic complexity of changes in disease or biological indicators. In recent years, a number of researchers have devoted their research to the multivariate joint model. For example^17–19^ are multivariate joint model investigations with continuous response variables. There are also many articles focusing on other data types, e.g. article^20^ considers multivariate binary data, the model in Rizopoulos and Ghosh^21^ combines continuous response variables with binary counterparts and article by Wang et al.^22^ uses ordinal data. Rue et al.^23^ build a joint multivariate model related to discrete data. Although the responses in Rue et al.^23^ are discrete variables, the longitudinal data model in the article still uses the normal distribution model and the beta model. Many software packages also provide relevant functionality for multivariate joint models. Examples include *merlin* in *Stata*^24^ and the *JMbayes* package in *R*.^25^ In this paper, we construct a bivariate discrete joint model to address the potential estimation bias caused by separate modelling and to avoid the lack of information caused by dropping longitudinal responses when using a univariate joint model. This joint model can take into account discrete data.

A joint model for longitudinal responses describes the change of responses using a model for longitudinal data and describes the risk of the event using a survival model.^26^ The principle of joint models is to link these two models using a common latent variable. This common latent variable captures the association between the processes. Based on this principle, two joint models have been proposed: the shared random effects model^11^ and the latent class model.^27^ Articles Rouanet et al.,^28^ Proust-Lima et al.^26^ and Proust-Lima et al.^29^ used latent class models to construct multivariate joint models. The limitation of the latent class model is that the classification may not be interpretable because we do not know what the different classes represent; moreover, we can only calculate the probability that an individual belongs to a certain class and cannot assign him or her to a specific class with certainty. To avoid these limitations, we model the latent variable as a random effect. Conditional on random effects we assume that the longitudinal outcome is independent of the time-to-event outcome.^14^ Also, we assume that the repeated measurements in the longitudinal outcome are independent of each other given the random effects.

A multivariate shared random effects model was introduced by Rizopoulos in Rizopoulos.^14^ In this model, the association between longitudinal variables is captured by the random effects. Our bivariate joint model uses an extended bivariate binomial distribution^30^ in the longitudinal part of the model. This distribution includes a parameter that represents the correlation coefficient between the two longitudinal outcomes. The model allows us to avoid using random effects to capture all the correlations of the joint model.

In this paper, we will investigate the bivariate responses changing over time, in which the responses are different measurements representing cognitive function for individuals. The shared random effects can take into account unobservable individual-specific characteristics.^14^ Since the cognitive function is usually measured by non-negative integers, the bivariate extension of the binomial distribution proposed by Altham and Hankin^30^ is used for the longitudinal model. This model not only measures whether the model is over- or underdispersed compared to the standard binomial distribution but also measures the association between the two longitudinal outcomes. For the survival part, the Weibull hazard model and the Gompertz hazard model are used. In univariate joint models, the link functions which contains in both the longitudinal model and the survival model are well defined.^31^ However, for the bivariate joint model, we do not know beforehand which link will provide the best result. We will therefore discuss several different link functions.

We apply the models to analyze the ELSA data. There are two non-negative longitudinal outcomes in the ELSA data, and they are related to verbal learning and recall. Individuals were asked to learn ten words and recall them at two different time points (immediate and later), but in the same interview; see Van den Hout and Muniz-Terrera.^7^ In the ELSA data, the event time for the joint model is the time of death.

The ‘Models section provides a brief overview of the models in this paper: the extension of the bivariate binomial distribution proposed by Altham and Hankin, the Weibull hazard model and the Gompertz survival model. The link between the longitudinal model and the survival model is also discussed in this part. The ‘Maximum likelihood function given left truncation section constructs the marginal log-likelihood function for the joint model, discusses the left truncation and proposes the method for computation. In the ‘Simulation section, we use a simulation study to validate the accuracy of the joint model parameter estimates. We analyze the ELSA data through fitting the shared random-effects model in the ‘Application section. The conclusion of this paper and possible extensions options for the joint model are discussed in the ‘Conclusion section.

## 2 Models

The joint model is given by

(1) $$\begin{array}{cc} \;p\left(y,t,t_{last}\right) & =\int p\left(y|t,b\right)p\left(t_{last}|b\right)p(b)db \end{array}$$

where y represents the longitudinal outcomes, t represents the corresponding time points, $t_{last}$ is the last recorded time and b represent the random effects. Distribution p(y|t,b) is the longitudinal model, $p(t_{last}|b)$ is the survival model and b represent the random effects.

The first part of this section introduces the bivariate binomial distribution used for the longitudinal model. The second part proposes the hazard survival model. The third part defines the link between the longitudinal model and the survival model, which consists of parameters common to both models and the random effects. For what follows, assume that the random effects are given by vector $b\in R^{p}$, where p is determined by the model definition. We will use p(b) to represent the distribution of random effects.

### 2.1 Longitudinal model

Generally, the scores for cognitive function are discrete non-negative integers. For example, the Mini-Mental State Examination (MMSE) test is a 30-point questionnaire, widely used extensively in clinical and research settings to measure cognitive impairment. A higher score represents better cognitive function. We model the probability of success (answering the question correctly and earning one point). For the MMSE, this would imply the assumption that the 30 questions are independent of each other and that the distribution of the total-score follows the binomial distribution. It is worth to notice that even if the probability of success is different, the total-score distribution still follows the binomial distribution.^32^

Let Y denote the response and p denote the probability of success. Parameter m represents the number of trials. Altham^33^ introduced the following extension of the binomial distribution.

(2) $$p(Y=y)=\frac{\left(\begin{array}{c} m\\ y \end{array}\right)p^{y}(1-p{)}^{\left(m-y\right)}\theta^{y(m-y)}}{C_{uni}}$$

for y∈{0,1,…,m}, where 0<p<1, θ>0, and

(3) $$C_{uni}=\sum_{y=0}^{m}\left(\begin{array}{c} m\\ y \end{array}\right)p^{y}(1-p{)}^{\left(m-y\right)}\theta^{y(m-y)}$$

When θ>1, distribution (2) is under-dispersed compared to the standard binomial distribution; when θ<1, the distribution is over-dispersed relative to the standard distribution.

In this paper, we use the bivariate extension of (2)^30^ to analyze the longitudinal data. Let $Y_{1}$ and $Y_{2}$ define the bivariate responses.

(4) $$p(Y_{1}=y^{\left(1\right)},Y_{2}=y^{\left(2\right)})=\frac{g(Y_{1}=y^{\left(1\right)})g(Y_{2}=y^{\left(2\right)})\phi^{y^{\left(1\right)}y^{\left(2\right)}}}{C_{biv}}$$

for $y^{\left(j\right)}\in \{0,1,\ldots ,m_{j}\}$, j=1,2.

(5) $$g\left(Y_{j}=y^{\left(j\right)}\right)=\left(\begin{array}{c} m_{j}\\ y^{\left(\;j\right)} \end{array}\right)p_{Y_{\;j}}^{y^{\left(j\right)}}(1-p_{Y_{\;j}}{)}^{\left(m_{j}-y^{\left(j\right)}\right)}\theta_{Y_{\;j}}^{y^{\left(j\right)}(m_{j}-y^{\left(j\right)})}$$

where $p_{Y_{j}}$ denote the probability of success for j=1,2; $0<p_{Y_{1}},p_{Y_{2}}<1$, $\theta_{Y_{j}},\phi >0$. The parameter ϕ is used to measure the correlation between two responses. When 0<ϕ<1, there is a negative correlation between two responses; when ϕ is greater than 1, there is a positive correlation between responses. The denominator in Equation (4) is:

$$C_{biv}=\sum_{y^{\left(1\right)}=0}^{m_{1}}\sum_{y^{\left(2\right)}=0}^{m_{2}}g(Y_{1}=y^{\left(1\right)})g(Y_{2}=y^{\left(2\right)})\phi^{y^{\left(1\right)}y^{\left(2\right)}}$$

Integers $m_{1}$ and $m_{2}$ for longitudinal responses $y^{\left(1\right)}$ and $y^{\left(2\right)}$ can be defined separately.

Assume that the random variable $b=(b_{10},b_{11},b_{20},b_{21}{)}^{T}$ includes the random intercepts ( $b_{10}$ and $b_{20}$) and the random slopes ( $b_{11}$ and $b_{21}$). Parameters $b_{\;j0}$ and $b_{\;j1}$ denote the corresponding random effects for $Y_{j},j=1,2$. Let t represent time or age.

To investigate the relationship between time and the response variables, we use the random-effects logistic regression model:

(6) $$p_{Y_{\;j}}=\frac{\exp\;\left((\eta_{0}^{\left(j\right)}+b_{\;j0})+(\eta_{1}^{\left(j\right)}+b_{\;j1})t\right)}{1+\exp\;\left((\eta_{0}^{\left(j\right)}+b_{\;j0})+(\eta_{1}^{\left(j\right)}+b_{\;j1})t\right)}$$

where ηs are the fixed effects in the model. We could add other covariates to model (6), such as sex and education level, to investigate the effect of these covariates on an individual’s cognitive function. We use x to denote any covariate, and the extension of model (6) containing a covariate could be written as:

(7) $$p_{Y_{\;j}}=\frac{\exp\;\left((\eta_{0}^{\left(j\right)}+b_{\;j0})+(\eta_{1}^{\left(j\right)}+b_{\;j1})t+\gamma_{L}^{\left(j\right)}x\right)}{1+\exp\;\left((\eta_{0}^{\left(j\right)}+b_{\;j0})+(\eta_{1}^{\left(j\right)}+b_{\;j1})t+\gamma_{L}^{\left(j\right)}x\right)}$$

the parameter $\gamma_{L}^{\left(j\right)}$ is the coefficient of the corresponding covariate and the corner label L represents the longitudinal model.

In this paper, we will investigate restricted models. We will explain the reasons for using restricted models in the later section. The restricted models are defined by restrictions on random effects, for example:

(8) $$b_{10}=b_{20},b_{11}=b_{21}$$

where two responses $Y_{1}$ and $Y_{2}$ share the same random intercept $b_{10}$ and the same random slope $b_{11}$.

We can also set

(9) $$b_{11}=b_{21}=0$$

which means that we only use random intercept for each response. Also, we can assume

(10) $$b_{10}=b_{20}=0$$

where responses have different random slope ( $b_{11}$ and $b_{21}$) and do not have corresponding random intercepts.

The reason for choosing this bivariate binominal distribution is that the formula of the probability density function is relatively simple, and it belongs to the exponential family.^30^ Moreover, this distribution allows $Y_{1}$ and $Y_{2}$ to have separate choices of $m_{1}$ and $m_{2}$.

### 2.2 Hazard models

We define the hazard models and the corresponding survivor models for analyzing the time-to-event data. The hazard model is a parametric regression model given by

(11) $$h(t)=h_{0}(t)\exp\;\left(\Delta (\alpha ,\eta ,b,\gamma_{S},t)\right)$$

where $h_{0}(t)$ is the baseline hazard function. Function Δ is a function of random effects, additional parameter α and common parameters. We define function Δ as the ‘shared part’ (link function) in the joint model, and it will be introduced in the next section. Vector $\eta =(\eta_{0}^{\left(j\right)},\eta_{1}^{\left(j\right)})$ represents the fixed parameters. Similar to equation (7), $\gamma_{S}$ is the parameter of the covariate and the corner label S represents the survival model. In the application in the ‘Simulation section, time t in the survival model is the last recorded age.

There are many widely used parametric hazard models in survival analysis, such as the exponential hazard model, the Weibull hazard model, the Gompertz hazard model, and the log-logistic distribution. In this paper we use two commonly adopted models to construct the survival part of the joint model: the Weibull hazard model and the Gompertz hazard model.

The Weibull model is a survival model with two positive parameters and it is a generalization of the exponential model. We denote the Weibull model Weibull(λ,τ). The shape of the function can be changed by varying the value of τ. In particular, when τ equal to 1 we obtain an exponential distribution. Xian Liu has mentioned that of all the families of parametric time distributions, the Weibull model is probably the most widely used parametric function in survival analysis due to its simplicity and flexibility,^34^ therefore we have chosen it as the first survival model used in this paper. The Gompertz model is the most widely used survival function to quantitatively describe human mortality and survival.^35,34^ The numerical estimation for the Gompertz model is computational robust compared with the Weibull model because there is no power function in the Gompertz model. Given that the event of interest is death, we have chosen the Gompertz model as the second survival model. Although in this paper we restrict the survival model to the Weibull model and the Gompertz model, the survival part of our joint model could be easily replaced by other frameworks.

We have the following baseline hazard models:

(12) $$\begin{array}{c} \text{Weibull}\;:\;h_{0}(t)=\lambda \tau t^{\tau -1}\\ \text{Gompertz}\;:\;h_{0}(t)=\lambda \exp\;\left(\xi t\right) \end{array}$$

where λ,τ>0, and the corresponding survivor functions are:

(13) $$\begin{array}{c} \text{Weibull}\;:\;p(T\geq t)=S(t)=\exp\;\left(-\lambda t^{\tau }\right)\\ \text{Gompertz}\;:\;p(T\geq t)=S(t)=\exp\;\left(-\frac{\lambda }{\xi }(\exp\;(\xi t)-1)\right) \end{array}$$

For computational reasons, we use the parametrization λ=exp(β) in the joint model to ensure the hazard is positive for any β∈R.

The definition for the range of ξ in the Gompertz model varies in the literature. Some researchers define ξ∈R, to ensure that the hazard exponentially increases over time when ξ>0 and decreases when ξ<0.^36^ However, if ξ<0 and t→∞, we have the following property of the survivor function:

(14) $$\lim_{t\to \infty }S(t)=\exp\;\left(\frac{\lambda }{\xi }\right)>0$$

The above equation shows that when the time is extremely large, the probability of survival is still larger than 0. It means that the event of interest does not occur for a proportion of the population. The events of interest in this paper are death, and the process from health to event occurring is irreversible. Therefore, we follow the definition given by Wienke^37^ and restrict ξ to be positive. In this case, when t=0 the hazard function equals to λ, and it increases to ∞ when t=∞. The corresponding survivor function equals to 1 when t=0, and it decreases to 0 when t goes up to ∞.

### 2.3 Link between the models

In this part, we introduce the link function $\Delta (\alpha ,\eta ,b,\gamma_{S},t)$ of the model, defined as an expression containing the random effects and the corresponding parameters. Parameters in this part are included in both the longitudinal model and the survival model. Based on the link function, we will make some remarks for the corresponding hazard models. Since in this section, we will focus on the structure of the random intercept and random slope, we will simplify the link function $\Delta (\alpha ,\eta ,b,\gamma_{S},t)$ to Δ(α,η,b,t).

The hazard model (11) allows for several specializations. In joint models, Δ(α,η,b,t) is often specified as $\alpha (\eta_{0}+b+(\eta_{1}+b)t)$ for the univariate model.^31^ In many articles related to multivariate joint models, the linear predictor is also commonly used as the link function. In our paper, we also use the linear predictor as the link function. However, when investigating the bivariate joint model, we do not know beforehand which kind of link will provide the best result. As we have mentioned before, we will use the restricted model based on equations (8) to (10) and construct the corresponding link function. We use restricted models for two reasons. Firstly, if we use the full model, i.e. models containing equations (6) or (7) for j=1,2, it would be unclear how to construct the link between the models and how to interpret the impact of the corresponding parameters on the risk of death. Secondly, we encountered challenges in computing high-dimensional integrals. Therefore, we reduced the dimensionality of the integrals, i.e. the number of corresponding random effects, while retaining the study of response-specific random effects (each response variable has a corresponding random effect). We consider a range of definitions of Δ, starting with

(15) $$\Delta (\alpha ,\eta ,b,t)=\alpha \left(\eta_{0}^{\left(j\right)}+b_{\;j0}\right)$$

where $\eta_{0}^{\left(j\right)}+b_{\;j0}$ is the random intercept for response $Y_{j}$, for j=1 or 2. We can also include the random intercept for both $Y_{1}$ and $Y_{2}$:

(16) $$\Delta (\alpha ,\eta ,b,t)=\sum_{\;j=1}^{2}\alpha_{j}\left(\eta_{0}^{\left(j\right)}+b_{\;j0}\right)$$

The advantage of (15) and (16) is that they are computationally easy because the link part is not affected by time. However, comparing with the value of the cognitive function at the start of the time scale, change in cognitive ability over time might have a greater impact on the risk of death or dementia. Therefore, we can include the random slopes in the same way.

(17) $$\Delta (\alpha ,\eta ,b,t)=\sum_{\;j=1}^{2}\alpha_{j}\left(\eta_{1}^{\left(j\right)}+b_{\;j1}\right)t$$

For the above link function containing random slopes, we also consider the restricted version, i.e. setting one of the $\alpha_{j}$s to 0

(18) $$\Delta (\alpha ,\eta ,b,t)=\alpha \left(\eta_{1}^{\left(j\right)}+b_{\;j1}\right)t$$

Equation (17) takes into account the trajectory of $Y_{1}$ and $Y_{2}$ changing over time by adding the fixed slope and the random slope. We can also use the random intercept and the random slope at the same time.

(19) $$\Delta (\alpha ,\eta ,b,t)=\sum_{\;j=1}^{2}\alpha_{1j}\left(\eta_{0}^{\left(j\right)}+b_{\;0}\right)+\alpha_{2j}\left(\eta_{1}^{\left(j\right)}+b_{\;1}\right)t$$

where the random effects are intercept/slope specific, and $Y_{1}$, $Y_{2}$ share the same random effects. We wish to distinguish the effect of the two response variables on the risk, therefore models with link function (19) will not be included in the application part of the research.

In addition to the link functions above, there are many other common forms of link functions. For example, in many joint models which use a linear mixed model as their longitudinal model, the linear predictor is the conditional expectation of the response variables. Such models make it possible to use the expectation in constructing the link function:

(20) $$\Delta (\alpha ,\eta ,b,t)=\alpha_{\;j}\left(\eta_{0}^{\left(j\right)}+b_{\;j0}+(\eta_{1}^{\left(j\right)}+b_{\;j1})t\right)$$

Equation (20) is a common form of using expectation $E(Y_{j})$ when constructing a link function. However, in our distribution, the expectation of the response is not the linear predictor. The expression in our joint model is a function of the range of the response m and the probability of success $p_{Y_{j}}$.

When substituting the above Δ expressions into the hazard models, we need to pay attention to the parameters λ , τ and ξ in the corresponding hazard functions.

For the Weibull hazard, when the expression of Δ is equal to (15) or (16). The hazard model can be written as:

(21) $$h(t)=\lambda^{*}\tau t^{\left(\tau -1\right)}$$

where the scale parameter λ is now written as an individual-specific parameter:

(22) $$\lambda^{*}=\exp\;\left(\beta +\alpha (\eta_{0}^{\left(j\right)}+b_{\;j0})\right)$$

The Weibull hazard model can also be written as a hazard model with an individual-specific slope parameter:

(23) $$h(t)=\lambda \tau^{*}t^{\left(\tau^{*}-1\right)}$$

where

(24) $$\tau^{*}=\exp\;\left(\alpha (\eta_{0}^{\left(j\right)}+b_{\;j0})\right)\tau$$

In this paper, we choose equation (21) as the corresponding hazard model. When the Δs include the random slopes (equations (18) and (19)) are substituted into Weibull hazard model, the shape parameter τ becomes a time-dependent parameter, which violates the definition of the standard Weibull model. When we include random slopes in the Weibull model, we have to calculate the log cumulative hazard function.^38^ In this paper, we will not discuss this case of using the log cumulative hazard function.

For the Gompertz hazard, we have:

(25) $$h(t)=\lambda^{*}\exp\;(\xi^{*}t)$$

where

(26) $$\begin{array}{c} \lambda^{*}=\exp\;\left(\beta +\alpha (\eta_{0}^{\left(j\right)}+b_{\;j0})\right)\\ \xi^{*}=\alpha (\eta_{1}^{\left(j\right)}+b_{\;j1})+\xi \end{array}$$

Both $\lambda^{*}$ and $\xi^{*}$ are individual-specific parameters. The Gompertz hazard model can easily handle the function Δ with random slopes. Because the model conditional on random effects is still a Gompertz hazard model.

### 2.4 Distribution of random effects

As we mentioned at the beginning of the ‘Models section, we use p(b) to represent the distribution of random effects. In this paper, we use the bivariate normal distribution as the distribution of random effects, i.e. $b∼\left(0,\left(\begin{array}{cc} \sigma_{b1}^{2} & \rho \sigma_{b1}\sigma_{b2}\\ \rho \sigma_{b1}\sigma_{b2} & \sigma_{b2}^{2} \end{array}\right)\right)$. Parameter ρ represents the correlation between random effects. The parameters $\sigma_{b1}$ and $\sigma_{b2}$ denote the standard deviation of the corresponding random effects respectively.

## 3 Maximum likelihood function given left truncation

Left truncation, also called *delayed entry*, occurs when individuals have been at risk before entering the study.^37^ For example, when the event of interest is death, individuals who died before the study started will not be included in the study. In ageing research, the main timescale is age. Since the event of interest is death, individuals can be included in the data only if they have not experienced the event before they enter the study. If we do not deal with the left truncation, the estimation is based on the assumption that individuals were not at risk of dying before the start of the study. Therefore, the left truncation needs to be taken into account in the model estimation.

For individual i, i=1,…,N, the corresponding longitudinal responses are $y_{i}=(y_{i}^{\left(1\right)},y_{i}^{\left(2\right)})$. Response $y_{i}^{\left(j\right)}$ is a vector $\left(y_{i}^{\left(j\right)}=(y_{i1}^{\left(j\right)},\ldots ,y_{in_{i}}^{\left(j\right)})\right)$ at age $t_{i}=(t_{i1},\ldots ,t_{in_{i}})$, where j=1,2 is the jth method of measuring cognitive function, $n_{i}$ is the number of observations for each individual. Let ω represent all the parameters in the joint model except the random effects. Let $t_{i1}$ denote baseline age. The likelihood contribution of individual i conditionally on truncation time $t_{i1}$ is:

(27) $$\begin{array}{rl} L_{i}(\omega |y_{i},t_{i},T\geq t_{i1}) & =p(y_{i},t_{i}|T\geq t_{i1},\omega )\\ & =\frac{\;p(y_{i},t_{i}|\omega )}{p(T\geq t_{i1}|\omega )} \end{array}$$

where $p(T\geq t_{i1}|\omega )$ is the survivor function evaluated at $t_{i1}$. For the shared random-effects model, the denominator in (27) can be written as:

$$p(T\geq t_{i1}|\omega )=\int p(T\geq t_{i1}|b_{i},\omega )p(b_{i}|\omega )db_{i}$$

Assuming independence between responses given the random effect, the marginal likelihood function is:

(28) $$p(y_{i},t_{i}|\omega )=\int p\left(y_{i}|t_{i},b_{i},\omega \right)p\left(t_{in_{i}}|b_{i},\omega \right)p\left(b_{i}|\omega \right)db_{i}$$

where $t_{in_{i}}$ is the last recorded age. Parameter $\delta_{i}=0$ means alive at the last observation and $\delta_{i}=1$ means death. Distribution $p(y_{i}|t_{i},b_{i})$ is defined by the longitudinal model and $p(t_{in_{i}}|b_{i},\delta_{i})$ by the survival model:

$$p\left(t_{in_{i}}|b_{i},\delta_{i}\right)=h{\left(t_{in_{i}}|b_{i}\right)}^{\delta_{i}}p(T\geq t_{in_{i}})$$

We define the random effect $b_{i}\in R^{p}$ by $b_{i}∼N(0,\sum )$, where ∑ is a p×p covariance matrix.

This model offers a flexible way to model the correlation between the value change of responses and the risk of event.^39^ The advantage of using the normal distribution is that it is one of the most common distributional choices in linear mixed-effects models and that it enables using Gauss-Hermite quadrature in the numerical optimization of the marginal likelihood. However, even if we can use the Gauss-Hermite integration, this model is still computationally intensive due to the numerical integration.

We code the marginal log-likelihood function in the *R* software.^40^ The corresponding parameters are estimated using the *ucminf* function in package *ucminf*.^41^ For the shared random-effects model (28), where the random effect b follows multivariate normal distribution, we use the Gauss-Hermite method with 7 nodes to do numerical integration. The accuracy of the node number selection will be verified in the ‘Simulation section.

It is worth mentioning that the bivariate Gauss-Hermite quadrature can be undertaken by using two univariate normal distributions (see, e.g. Van Den Hout^39^). The bivariate normal distribution f(z,x) can be expressed as:

(29) $$f_{Z|X}(z|X=x)f_{X}(x)$$

where $Z∼N(\mu_{Z},\sigma_{Z}^{2})$, $X∼N(\mu_{X},\sigma_{X}^{2})$ and $Z|X∼N(\mu_{Z}-\rho (\sigma_{Z}/\sigma_{X})x-\mu_{X},\sigma_{Z}^{2}(1-\rho^{2}))$. Parameter ρ represents the correlation between Z and X.

## 4 Simulation

We conduct a small simulation study to investigate the parameter estimation for the joint model. We used the ADEMP structure to plan the simulation study.^42^ The ADEMP structure includes *Aims, Data-generating mechanisms, Methods, Estimands, Performance measures*.

The main *Aims* of our simulation study are to check the estimation method and to investigate the performance of joint model given various sample sizes.

*Date-generating mechanism:* We use the joint model with the best results in the ‘Application section as the model for the ‘Simulation section. The mechanism is based on the extension of the bivariate binomial distribution (4) and the Gompertz hazard model (13). As Morris et al.^42^ mentioned, varying the sample size of the simulation dataset is a common approach when changing the data generation mechanism since the performance tends to vary with the sample size. We also refer to the article by Van den Hout and Muniz-Terrera,^4^ which designed a small simulation study in a similar setting. Therefore, to investigate the small sample bias, for the joint model we set the sample size N=100,400,1000, respectively.

For the joint model, the logistic regression model is defined by (6) with $b_{11}=b_{21}=0$, with the corresponding link function (15) for j=1. The random-effects distribution for $\left(b_{10},b_{20}\right)$ is specified by $\sigma_{b1}$, $\sigma_{b2}$ and ρ. This joint model is the best performing model in the application; see the ‘Application section.

The true values of the parameters, i.e. the values used when generating the data, are listed as ‘Value’ in Table 1. It is worth noting that we set α to −0.4 in the model. The parameter α is the relevant parameter of the link function Δ(α,η,b,t), if α is significantly different from 0, it means that there is an association between the longitudinal model and the survival model, and it is necessary for us to build the joint model.

**Table 1.** Simulation study for the first joint model.The follow-up interval is 3.

		N=100						N=400						N=1000					
Parameter	Value	Mean	Bias	% bias	SE	MC.Bias	MC.SE	Mean	Bias	% bias	SE	MC.Bias	MC.SE	Mean	Bias	% bias	SE	MC.Bias	MC.SE
$\eta_{0}^{\left(1\right)}$	1.0	1.020	0.020	2.0	0.282	0.029	0.020	1.011	0.011	1.1	0.128	0.013	0.009	1.008	0.008	0.8	0.084	0.008	0.006
$\eta_{1}^{\left(1\right)}$	−0.2	−0.207	−0.007	3.5	0.046	0.005	0.003	−0.201	−0.001	0.7	0.020	0.002	0.001	−0.200	0.000	0.2	0.013	0.001	0.001
$\theta_{Y_{1}}$	1.0	1.009	0.009	0.9	0.041	0.004	0.003	1.002	0.002	0.2	0.019	0.002	0.001	1.001	0.001	0.1	0.011	0.001	0.001
$\eta_{0}^{\left(2\right)}$	0.8	0.821	0.021	2.6	0.295	0.030	0.021	0.817	0.017	2.1	0.142	0.014	0.010	0.809	0.009	1.2	0.090	0.009	0.006
$\eta_{1}^{\left(2\right)}$	−0.2	−0.202	−0.002	1.2	0.044	0.004	0.003	−0.201	−0.001	0.4	0.020	0.002	0.001	−0.200	0.000	0.2	0.013	0.001	0.001
$\theta_{Y_{2}}$	1.0	1.003	0.003	0.3	0.038	0.004	0.003	1.000	0.000	0.0	0.016	0.002	0.001	1.000	0.000	0.0	0.011	0.001	0.001
ϕ	0.8	0.798	−0.002	0.2	0.048	0.005	0.003	0.797	−0.003	0.3	0.022	0.002	0.002	0.798	−0.002	0.2	0.015	0.001	0.001
ξ	0.1	0.118	0.018	17.6	0.042	0.004	0.003	0.102	0.002	2.2	0.018	0.002	0.001	0.100	0.000	0.3	0.010	0.001	0.001
$\sigma_{b1}$	0.4	0.424	0.024	6.0	0.148	0.015	0.011	0.404	0.004	0.9	0.063	0.006	0.004	0.405	0.005	1.4	0.040	0.004	0.003
$\sigma_{b2}$	0.6	0.610	0.010	1.7	0.149	0.015	0.011	0.604	0.004	0.6	0.074	0.007	0.005	0.601	0.001	0.2	0.044	0.004	0.003
ρ	0.3	0.248	−0.052	17.5	0.393	0.040	0.028	0.308	0.008	2.6	0.173	0.017	0.012	0.315	0.015	5.0	0.113	0.011	0.008
β	−1.5	−1.500	0.000	0.0	0.603	0.062	0.044	−1.504	−0.004	0.3	0.254	0.025	0.018	−1.505	−0.005	0.4	0.145	0.015	0.010
α	−0.4	−0.453	−0.053	13.2	0.653	0.067	0.047	−0.410	−0.010	2.6	0.263	0.026	0.019	−0.399	0.001	0.1	0.146	0.015	0.010

MC.Bias: Monte Carlo standard error of bias; MC.SE: Monte Carlo standard error of empirical SE.

*Estimands:* In this simulation study, the estimands of interest are the model parameters over the $N_{sim}$ iterations.

*Methods:* In the simulation study, to eliminate the effect of left truncation, we set the observation time for all individuals beginning at time zero. If an individual does not drop out, the follow-up ends at 24 years. Follow-up intervals are set to three years to ensure that we have enough information about changes in response variables over time. When the follow-up interval was fixed at once every three years, it means that there are 9 observations for individuals who do not drop out at the end of the experiment.

Given the specified parameters and follow-up intervals, the data for an individual is firstly simulated by drawing random effects. Based on these effects, the bivariate longitudinal trajectories were simulated using the Metropolis-Hastings algorithm. Afterwards, random effects are used again to define the Gompertz parameter $\lambda^{*}=\exp\;(\beta +\alpha (\eta_{0}^{\left(1\right)}+b_{10}))$ for the first joint model and to calculate the survival function S(t) for each follow-up time point. Using the cumulative distribution function F(t)=1−S(t), the time point at death is derived by using the inversion method. When estimating the parameters, we use Gauss-Hermite quadrature to calculate the double integral. We choose 7 nodes for the corresponding Gauss–Hermite quadrature. The corresponding parameters are estimated using the *ucminf* function in package *ucminf*.^41^ The number of iterations is 100.

*Performance measures:* In this section, we will calculate the means of estimated parameters over the $N_{sim}$ iterations. We will assess means, bias, relative bias (%), empirical standard error (SE), Monte Carlo SE of bias (MC.Bias) and Monte Carlo SE of empirical SE (MC.SE).^42^ Bias and relative bias are the main performance measures we are interested in. Monte Carlo SE is a statistic that quantifies the uncertainty of a simulation study with finite $N_{sim}$.

Table 1 shows the simulation results for the choices N=100,400,1000, with follow-up intervals equals three. The number of iterations is 100. As we can observe in Table 1, in general, the mean of estimated values over the 100 iterations deviates little from the true values we set. The relative biases for most parameters are below 5%. It is worth noting the estimated values of the parameter ξ of the Gompertz hazard model, the correlation coefficient ρ of the random effects and the estimated value of the corresponding parameter α of the link function Δ(α,η,b,t). Although the estimates of these parameters have non-negligible biases when the sample size is small (the relative bias of these three parameters is 17.6%, 17.5% and 13.2%, respectively when the N=100), the relative biases for all parameters are quite close to zero when the sample size is expanded to 1000. Overall, the mean of the estimated value will be closer to the set value when N is increased. As the sample size increases, there is a significant improvement in other relevant estimates such as bias, relative bias and SE.

**Figure 1.**
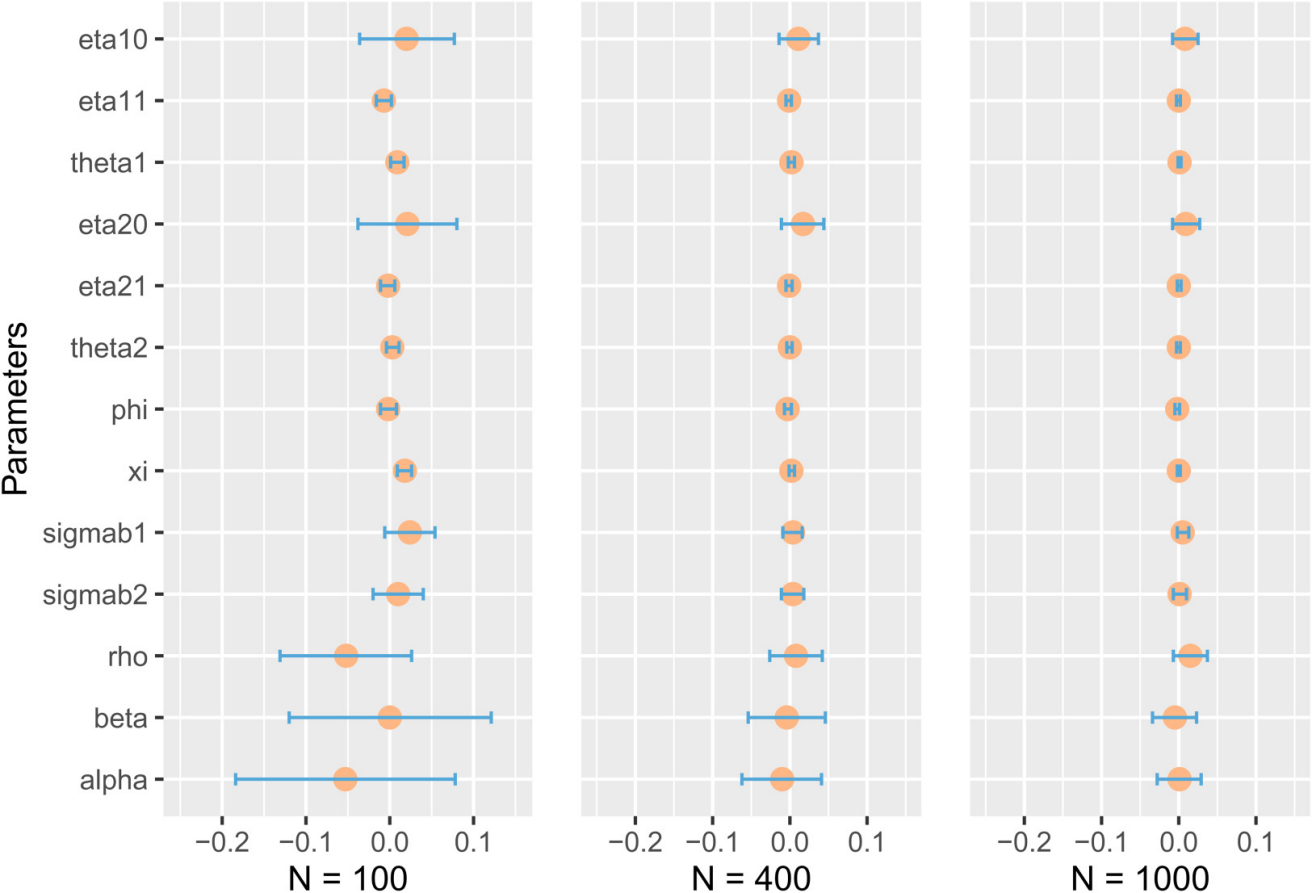
Bias and corresponding Monte Carlo 95% confidence interval. Circles represent biases, and geometry bars represent Monte Carlo 95% confidence interval.

Based on the bias values and the corresponding MC.Bias values in Table 1, we plot the Figure 1. From this figure, we can observe that for most of the parameters, 0 is included in the 95% confidence interval. From the left to the right of the figure, i.e. from N=100 to N=1000, the corresponding confidence intervals for the parameters’ biases show a clear reduction. This means that the larger the sample size the closer the mean is to the true value, which is consistent with our previous discussion on Table 1.

This simulation also shows that

1. The estimation by marginal likelihood can reproduce the parameters that were used to generate the data.
2. The function *ucminf* combined with the Gauss-Hermite method gives accurate estimates. The settings of the arguments in this paper are reasonable, e.g. in the Gauss-Hermite method the node is set to 7 and in the simulation the number of iterations is set to 100. It is worth noting that while in this paper we have justified setting the number of nodes to 7 through simulation, in other cases, such as when there is considerable heterogeneity between subjects, we may need more nodes when using the Gauss-Hermite method.
3. The bias of the parameter estimates decreases significantly when the sample size increases. In particular, when the sample size is N=1000, 0 is included in the 95% confidence interval of the estimated biases of all parameters. This result also justifies our choice of a sample size of 1000 in the ‘Application section.

## 5 Application

The ELSA is a rich resource of information on the dynamics of health, social, wellbeing and economic circumstances in the English population aged 50 and older. Established in 2002, the original sample was drawn from households that had previously responded to the Health Survey for England between 1998 and 2001. The same group of respondents have been interviewed at two-yearly interviews, known as ‘waves’, to measure changes in their health, economic and social circumstances. Younger age groups are replaced or refreshed to retain the panel. Data from the ELSA can be obtained via the Economic and Social Data Service (www.esds.ac.uk). The information collected provides data on household and individual demographics, health, social care, work and pensions, income and assets, housing, cognitive function, social participation, effort and reward, expectations, walking speed and weight for individuals.

In this paper, we focus on a test with two responses in the ELSA data.^43^ This test asks individuals to remember 10 words in the same interview. The first response in the test, representing the individual’s short-term memory ability to some extent, is the number of words the individual recalls immediately (immediate recall). An individual’s long-term memory ability is indicated by recording the number of words he/she can remember after five minutes (delayed recall).

The number of immediate-recall words is represented by $Y_{1}$ and that for the delayed-recall words is $Y_{2}$, in which $Y_{j}\in \{0,1,\ldots ,10\},j=1,2$. The corresponding time (age) is given in integers for the reason of confidentiality.^43^

In the ELSA data, there are 11,932 individuals interviewed. In this section, we analyze the ELSA data which were also used in Van den Hout and Muniz-Terrera’s paper.^4^ Van den Hout and Muniz-Terrera processed the data via the following four aspects: remove individuals who were interviewed only once with missing data on the number of words; remove individuals without information on the year of birth; remove individuals who were younger than 50 years old at baseline wave 1 and remove individuals with censored age at baseline.

In this paper, the main purpose of this paper is to illustrate the methodology rather than a study of ELSA data. Therefore, we use a subset of the data above.^7^ The subset has N=1000 individuals, randomly sampled from the full data conditional on two restrictions:

1. Individuals are younger than 90 years at baseline and do not have censored age during follow-up.
2. Each individual has at least two records. These two records can either include two observations or an observation and a time of death.

The age in this dataset is rescaled by subtracting 49. The number of deaths is 195, where the attrition rate is close to the full data. The ratio of women to men in the data is 540: 460, which is also close to that ratio for the full data. The average of first recorded immediate-recall words for all individuals is 5.60, and that for the delayed recall is 4.19. Other details for this subset can be found in Van Den Hout.^39^

**Figure 2.**
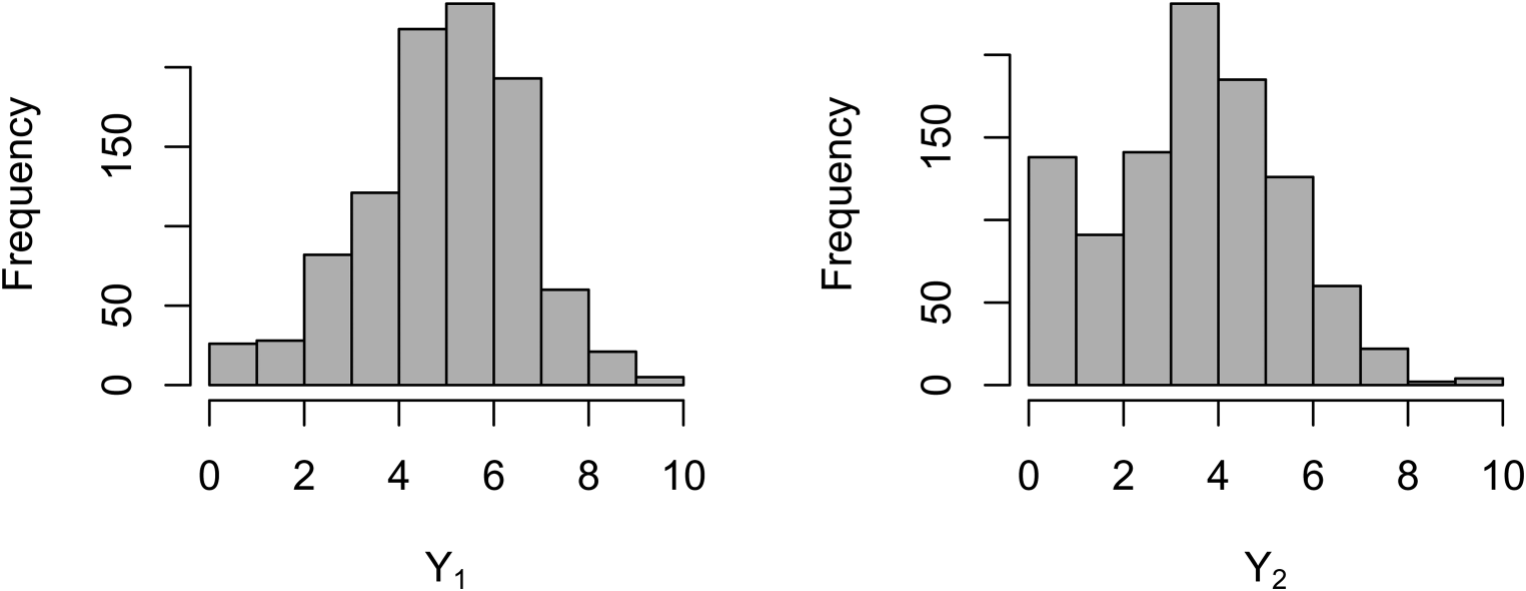
Frequency distribution of recalled words at baseline interview. $Y_{1}$ represents immediate-recall words; $Y_{2}$ represents delayed-recall words.

**Figure 3.**
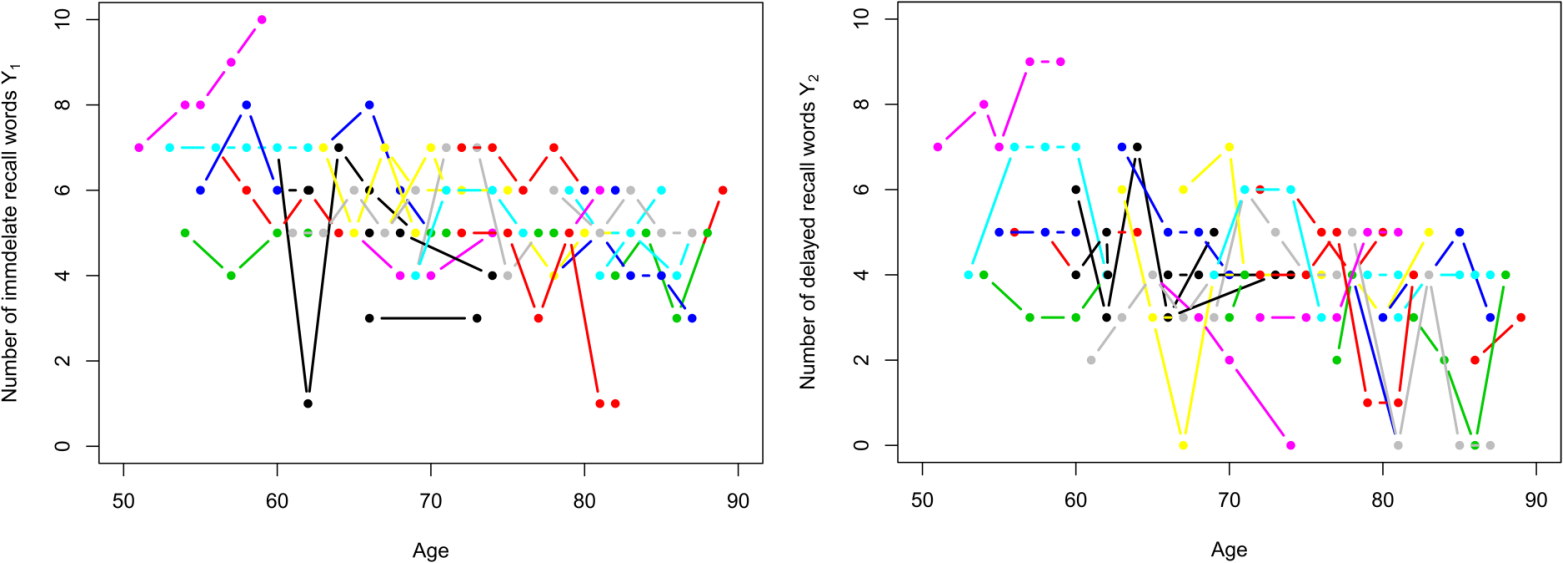
Recalled words trajectories. Left-hand side: immediate-recall words $Y_{1}$ trajectories. Right-hand side: delayed-recall words $Y_{2}$ trajectories. Individuals are represented in the same colour in both plots.

Figure 2 shows the frequency distribution of recalled words at baseline interview. Compared with the distribution of immediate recall words, the distribution of delayed-recall words is concentrated on the left. It can also reflect that individual’s short-term memory is better than long-term memory. In Figure 3, we select 30 individuals and plot the trajectories of their responses. We can see from the picture that an individual’s ability to remember words (cognitive ability) is fluctuating and overall the number of words that people remember decreases over time.

The joint model in this subsection uses the extension of bivariate binomial distribution mentioned in the the ‘Longitudinal model section; the Gompertz hazard model and the Weibull hazard model mentioned in the the ‘Hazard models section. The longitudinal model and the survival model are joined together given the normally distributed bivariate random effects. The joint model mentioned in this section is divided into two main parts: the first part is a bivariate extension model based on the univariate model proposed in Van den Hout and Muniz-Terrera’s paper^4^; the second part contains joint models constructed based on the link functions we discussed in the ‘Link between the models section.

### 5.1 Bivariate extension of joint model based on previous paper^4^

Van den Hout and Muniz-Terrera constructed a univariate shared random-effect joint model with binomial distribution and beta-binomial distribution.^4^ Here, we would like to use a similar method, i.e. a shared random-effect method, to construct bivariate joint models. Although we use different distributions and have different numbers of responses, because the same shared random-effect method is applied, we think it is still worthwhile to construct a bivariate model with a similar structure based on this univariate model in Van Den Hout and Muniz-Terrera.^4^ We use the same structure with random intercept and random slope as in Van Den Hout and Muniz-Terrera^4^ for one of the responses, and the other response is modelled with fixed effects.

For the univariate joint model in Van Den Hout and Muniz-Terrera^4^, the estimation of αs are negative. If the estimation of αs in the bivariate joint model is similar to the αs for the univariate joint model, ie. both αs are negative, we can infer that our method is consistent with the analysis based on the univariate model. Since the response variable of the univariate joint model is delayed recall $Y_{2}$, we add the random effects to the linear predictor associated with $Y_{2}$. We assume $p_{Y_{2}}$ equals to (6), where j=2. For immediate recall $Y_{1}$, we have the logistic regression model $p_{Y_{1}}$:

(30) $$p_{Y_{1}}=\frac{\exp\;(\eta_{0}^{\left(1\right)}+\eta_{1}^{\left(1\right)}t)}{1+\exp\;(\eta_{0}^{\left(1\right)}+\eta_{1}^{\left(1\right)}t)}$$

The corresponding link function is

(31) $$\Delta (\alpha ,\eta ,b,t)=\alpha_{1}\left(\eta_{0}^{\left(2\right)}+b_{20}\right)+\alpha_{2}\left(\eta_{1}^{\left(2\right)}+b_{21}\right)t$$

The hazard model also contains the same link function for delayed recall. The hazard model equals to (25). Parameter $\lambda^{*}$ and $\xi^{*}$ in the hazard model equal to (26) for j=2. Estimated result are provided in Table 2.

**Table 2.** ELSA: Parameter estimates for the bivariate extension of the joint model in paper.^4^ The values in parentheses are the standard errors of the corresponding parameters.

$\eta_{0}^{\left(1\right)}$	−0.857 (0.053)	$\theta_{Y_{1}}$	1.122 (0.009)	$\sigma_{b1}$	1.116 (0.073)	β	−6.800 (0.497)
$\eta_{0}^{\left(2\right)}$	−1.969 (0.094)	$\theta_{Y_{2}}$	1.167 (0.011)	$\sigma_{b2}$	0.055 (0.005)	ξ	0.097 (0.012)
$\eta_{1}^{\left(1\right)}$	−0.014 (0.002)	ϕ	1.432 (0.016)	ρ	−0.682 (0.190)		
$\eta_{1}^{\left(2\right)}$	−0.038 (0.004)			$\alpha_{1}$	−0.400 (0.168)	$\alpha_{2}$	−0.193 (0.086)

The αs’ estimates in Table 2 meet our expectation. The estimates ${\hat{\alpha }}_{1}=-0.400$ is negative and significantly different from zero, which means that the risk of death is relatively lower when the individual has a better cognitive function at the baseline age. Meanwhile, the parameter ${\hat{\alpha }}_{2}=-0.193$ is also negative, implying that the risk of death increases with time given that $b_{21}$ is equal to 0 on average.

The probability of remembering a word at baseline age ( t=0), conditional on random effects being equal to its mean 0, could be calculated via parameter $\eta_{0}^{\left(j\right)},j=1,2$. For the immediate-recall number, the corresponding we have ${\hat{\eta }}_{0}^{\left(1\right)}=-0.857$ and the corresponding probability equals to 0.298; for the number of delayed recall, the corresponding probability decreases to 0.122 with ${\hat{\eta }}_{0}^{\left(2\right)}=-1.969$. The probability of remembering a word immediately at baseline age is higher than that of recalling later. It means that the expected immediate-recall number is larger than the expected number of the delayed recall. Both ${\hat{\eta }}_{1}^{\left(1\right)}$ and ${\hat{\eta }}_{1}^{\left(2\right)}$ are smaller than 0, which shows that individuals’ memory or cognitive function declines with age. Moreover, since ${\hat{\eta }}_{1}^{\left(1\right)}>{\hat{\eta }}_{1}^{\left(2\right)}$, we can infer that for each individual the transient memory decays more slowly than long-term memory with age.

### 5.2 Bivariate extension joint models with different link functions Δ

We will now construct the joint model based on the link function mentioned in the ‘Link between the models section. Our joint model consists of the bivariate extension binomial distribution as the longitudinal model and the Gompertz hazard or the Weibull hazard as the survival model. Gender is added to the model as a covariate (0 for women, 1 for men). The corresponding logistic regression model and link function is given by

(32) $$p_{Y_{\;j}}=\frac{\exp\;\left((\eta_{0}^{\left(j\right)}+b_{\;j0})+(\eta_{1}^{\left(j\right)}+b_{\;j1})t+\gamma_{L}^{\left(j\right)}sex\right)}{1+\exp\;\left((\eta_{0}^{\left(j\right)}+b_{\;j0})+(\eta_{1}^{\left(j\right)}+b_{\;j1})t+\gamma_{L}^{\left(j\right)}sex\right)}$$

where j=1 or 2.

We also add gender to the link function $\Delta (\alpha ,\eta ,b,\gamma_{S},t)$. Taking equation (16) as an example, in this section we write equation (16) as:

(33) $$\Delta (\alpha ,\eta ,b,\gamma_{S},t)=\sum_{\;j=1}^{2}\alpha_{j}\left(\eta_{0}^{\left(j\right)}+b_{\;j0}\right)+\gamma_{S}sex$$

Because we want to distinguish the effect of two responses on the risk of death, in each joint model we set only one random effect for one response. It means that we only discuss the case where both responses have random intercepts or both have random slopes. Moreover, in the joint model, we parameterize the correlation between the two responses multiple times using four parameters: ϕ in bivariate extension binomial model (4), correlation ρ between corresponding random effects for two responses and link function parameters $\alpha_{1}$ and $\alpha_{2}$. We will discuss the case in which $\alpha_{1}$, $\alpha_{2}$ and ρ are equal to 0 respectively.

We use INT to denote the joint model containing random intercept in the link function, i.e. link function equals to (15) or (16). In this case for the longitudinal model we restrict $b_{11}=b_{21}=0$ (equation (9)). We use SLO to represent models with random slope in the link function. In this situation, the corresponding link function is equal to either (18) or (17), and the corresponding longitudinal are restricted to equation (10). The letters G and W denote models containing the Gompertz hazard model and the Weibull hazard model, respectively.

The Akaike information criterion (AIC) values and the corresponding parameters of link function Δ are presented in Table 3. Models INT.W1 - 4 and Model INT.G1 - 4 in Table 3 represent joint models with random intercept. For Model SLO.G1 - 4, we construct the joint model with random slope.

**Table 3.** ELSA: AIC and estimated αs for shared random-effects joint models. −2LL represents the -2loglikelihood.

MODEL	$p_{Y_{j}}$	Link function	Hazard distribution	−2LL	AIC	α	$\alpha_{1}$	$\alpha_{2}$	ρ
*INT.W*1		(16)	Weibull	100985.1	101019.1		−0.480	−0.031	0.316
*INT.W*2		(15) *j* = 1	Weibull	100985.2	101017.2	−0.508			0.318
*INT.W*3		(15) *j* = 2	Weibull	100989.2	101021.2	−0.154			0.327
*INT.W*4	(9)	(16)	Weibull	101001.2	101033.2		−0.507	−0.075	
*INT.G*1		(16)	Gompertz	100965.5	100999.5		−0.609	−0.007	0.315
*INT.G*2		(15) *j* = 1	Gompertz	100965.5	100997.5	−0.616			0.316
*INT.G*3		(15) *j* = 2	Gompertz	100971.2	101003.2	−0.168			0.329
*INT.G*4		(16)	Gompertz	100981.5	101013.5		−0.632	−0.065	
SLO.G1	(10)	(17)	Gompertz	103373.2	103407.2		0.062	−0.015	0.474
SLO.G2		(18) *j* = 1	Gompertz	101170.6	101202.6	−0.617			0.436
SLO.G3		(18) *j* = 2	Gompertz	101179.1	101211.1	−0.117			0.431
SLO.G4		(17)	Gompertz	101197.5	101229.5		−0.583	−0.069	

ELSA: English Longitudinal Study of Ageing; AIC: Akaike information criterion.

We expect the risk of death to be relatively lower when individuals have a good cognitive function at baseline age or the downward trajectory over time of cognitive function is slow. For all models except Model SLO.G1 in Table 3, the αs are estimated as smaller than 0. For Models *INT.W*1 - 4 and *INT.G*1 - 4, the negative αs mean that the risk of death is relatively lower when the individual has a better cognitive function at the baseline age. For Models SLO.G2, SLO.G3 and SLO.G4, the risk of death is lower when the cognitive function declines relatively slower for an individual.

When the link function has random slopes for both responses (17), the model’s estimate of $\alpha_{1}$ is not in line with our expectations with respect to the process. The estimated parameter ${\hat{\alpha }}_{1}=0.062$ in Model SLO.G1 is positive. This positive $\alpha_{1}$, representing the individual risk of death becomes smaller over time with the corresponding random slope of immediate recall. This result is the opposite of what we expected: instead of the risk of death increasing over time, it decreases over time.

In general, the AIC value for the model with random intercept (e.g. Model *INT.W*2) is smaller than that for the corresponding model with random slope (e.g. Model SLO.W2). It means that there was little heterogeneity in slope between subjects, so the increase in model likelihood was negligible compared to the increase in complexity.

- **Random intercept for both $Y_{1}$ and $Y_{2}$**
  The joint Model *INT.G*2, using the Gompertz survival model with link function (15) for j=1, fits the best. We present the parameter estimation in Table 4.
  In Table 4, we have ${\hat{\eta }}_{0}^{\left(1\right)}=-0.580$ is larger than ${\hat{\eta }}_{0}^{\left(2\right)}=-2.014$, we can calculate the corresponding probability of remembering a word at baseline age via these two parameters. The probability of recalling a word immediately at baseline age given b=0 is 0.359, in which is higher than that for recalling a word later (0.118). The high probability of remembering words implies better expected cognitive function. For an individual, the expected immediate-recall number is larger than that of the delayed recall. Meanwhile, the estimation of $\hat{\alpha }=-0.616$ is negative, which means that the risk of death is relatively lower when the individual has a better cognitive function. Based on the estimated SE of α, α is significantly different from zero. This implies that there is a link between the longitudinal process and the survival process. Moreover, both ${\hat{\eta }}_{1}^{\left(1\right)}$ and ${\hat{\eta }}_{1}^{\left(2\right)}$ are smaller than 0, which means that the probability of recalling a word decreases with age. We also notice that random effects $b_{1}$ and $b_{2}$ have a positive correlation with $\hat{\rho }=0.370$. It means that if the individual has great short-term memory (immediately recall ability) at the beginning of the research, the corresponding long-term memory (delayed recall ability) will be slightly positively affected. Parameters ${\hat{\theta }}_{Y_{1}}$ and ${\hat{\theta }}_{Y_{2}}$ are both larger than 1, which means that the estimated distribution is more peaked than the standard bivariate binomial distribution. The three parameters related to gender, ${\hat{\gamma }}_{L}^{\left(1\right)}$ and ${\hat{\gamma }}_{L}^{\left(2\right)}$ in the longitudinal model are both negative. This result means that, with other parameters being equal, men are expected to have lower cognitive function than women. Parameter ${\hat{\gamma }}_{S}=0.370$ implies a higher risk of death for men than for women under the same circumstances.
- **Additional remarks**
  Compare with the probability of remembering a word ( $\eta_{0}^{\left(1\right)}$ and $\eta_{0}^{\left(2\right)}$) at baseline level, we expect that change in cognitive ability over time will have a greater impact on the risk of death.
  For the Gompertz hazard model, we rewrite the hazard model based on (25):
(34) $$\begin{array}{c} h(t)=\exp\;\left(\beta +\eta_{0}^{\left(j\right)}+\alpha (\eta_{1}^{\left(j\right)}+b_{\;j1})t+\xi t\right)\\ \;\;=\exp\;\left\{\beta +\eta_{0}^{\left(j\right)}+\left(\alpha (\eta_{1}^{\left(j\right)}+b_{\;j1})+\xi \right)t\right\} \end{array}$$
  In this case, the parameter $\xi^{*}$ for the Gompertz model is:
(35) $$\xi^{*}=\alpha (\eta_{1}^{\left(j\right)}+b_{\;j1})+\xi .$$
  In Table 3, we constructed four models which use random slope as link function (Model SLO.G1 - 4). The joint Model SLO.G2, using the Gompertz survival model with link function (18) for j=1, has the best fitting effect among the four models.
  In Model SLO.G2, the estimation of ${\hat{\eta }}_{0}^{\left(1\right)}=-0.631$ is higher than ${\hat{\eta }}_{0}^{\left(2\right)}=-1.956$, representing the higher probability of remembering a word. The expected immediate-recall number for an individual is greater than that of the delayed recall. The estimation of $\hat{\alpha }=-0.617$ is negative, representing the relatively lower risk of death when the individual has a better cognitive function. Estimated ${\hat{\eta }}_{1}^{\left(1\right)}$ and ${\hat{\eta }}_{1}^{\left(2\right)}$ are smaller than 0, which means that the probability of recalling a word decreases with age. Similarly with Table 4, the correlation between random effects $b_{1}$ and $b_{2}$ is positive ( $\hat{\rho }=0.436$). This time, it shows that if an individual’s instant memory declines rapidly with age, his/her long-term memory may also decline fast.
  In Table 3, we have $\hat{\alpha }>$ 0 when we include random effects for both $Y_{1}$ and $Y_{2}$ in the survival model. These estimates represent that higher cognitive function leads to a high risk of death. The estimated αs are not affected by the type of random effects (intercept/slope). Although the AIC value for the joint model SLO.G1 is not the smallest, the values of ${\hat{\alpha }}_{1}$ is still worth to be analyzed. This estimation may be caused by the correlation between $Y_{1}$ and $Y_{2}$. In the model in this paper, we captured the relationship between the two responses several times via parameters ϕ, ρ and αs. Overfitting in this model may lead to unexpected results.

**Table 4.** ELSA: Parameter estimates for Model *INT.G*2 with (15) *j* = 1. The values in parentheses are the standard errors of the corresponding parameters.

$\eta_{0}^{\left(1\right)}$	−0.580 (0.080)	$\theta_{Y_{1}}$	1.206 (0.035)	$\sigma_{b1}$	0.447 (0.101)	β	−7.043 (0.350)
$\eta_{0}^{\left(2\right)}$	−2.014 (0.108)	$\theta_{Y_{2}}$	1.123 (0.028)	$\sigma_{b2}$	0.649 (0.094)	ξ	0.122 (0.009)
$\eta_{1}^{\left(1\right)}$	−0.024 (0.002)	ϕ	1.425 (0.061)	ρ	0.316 (0.082)		
$\eta_{1}^{\left(2\right)}$	−0.029 (0.003)					α	−0.616 (0.227)
$\gamma_{L}^{\left(1\right)}$	−0.063 (0.042)	$\gamma_{L}^{\left(2\right)}$	−0.130 (0.051)	$\gamma_{S}$	0.370 (0.148)		

### 5.3 Model fit

In order to investigate model fit, in this section we will first calculate the corresponding individual-specific random effects and then plot the fitted distribution based on the mean of random effects. After that we will discuss the accuracy of model predictions by comparing the predicted survival curves with the observed survival curves.

Denote the responses for individual i by ${\tilde{y}}_{i}$. Let ${\tilde{t}}_{in_{i}}$ denote the age corresponding to the last observed data, and ${\tilde{t}}_{i1}$ denote the age corresponding to the baseline age. When there is only one observation, we set ${\tilde{t}}_{in_{i}}={\tilde{t}}_{i1}$. In order to predict an individual trajectory given observed data, we need the corresponding values of random effects. We use Maximum a posterior (MAP) estimation to derive the most likely value of the random effects:

(36) $$\begin{array}{c} \;p(b_{i}|{\tilde{t}}_{i1},{\tilde{t}}_{in_{i}},\delta_{i}=0,{\tilde{y}}_{i};\omega =\hat{\omega })\propto p({\tilde{t}}_{in_{i}}|\delta_{i}=0,b_{i};\omega =\hat{\omega })p({\tilde{y}}_{i}|b_{i};\omega =\hat{\omega })p(b_{i}|\omega =\hat{\omega }) \end{array}$$

One disadvantage of shared random-effects model is that we define the distribution of random effect before fitting models. We can verify whether the distribution of random variables is the same as the preset distribution, which is bivariate normal distribution in this paper, based on the estimated random effects. The estimated random effects for model in the ‘Bivariate extension of joint model based on previous paper[4] section follows bivariate normal distribution $b_{i}∼N\left(\left(\begin{array}{c} -0.017\\ 0.002 \end{array}\right),\left(\begin{array}{cc} 0.306 & -0.345\\ -0.345 & 0.001 \end{array}\right)\right)$. The expectations of random effects are close to what we set, and the estimated variances are smaller that the result in Table 2. Based on Nan Laird’s book,^44^ this result is in line with our expectation. Given estimated random effects, both survival and longitudinal responses can be predicted given estimated random effects and an assumed age.

**Figure 4.**
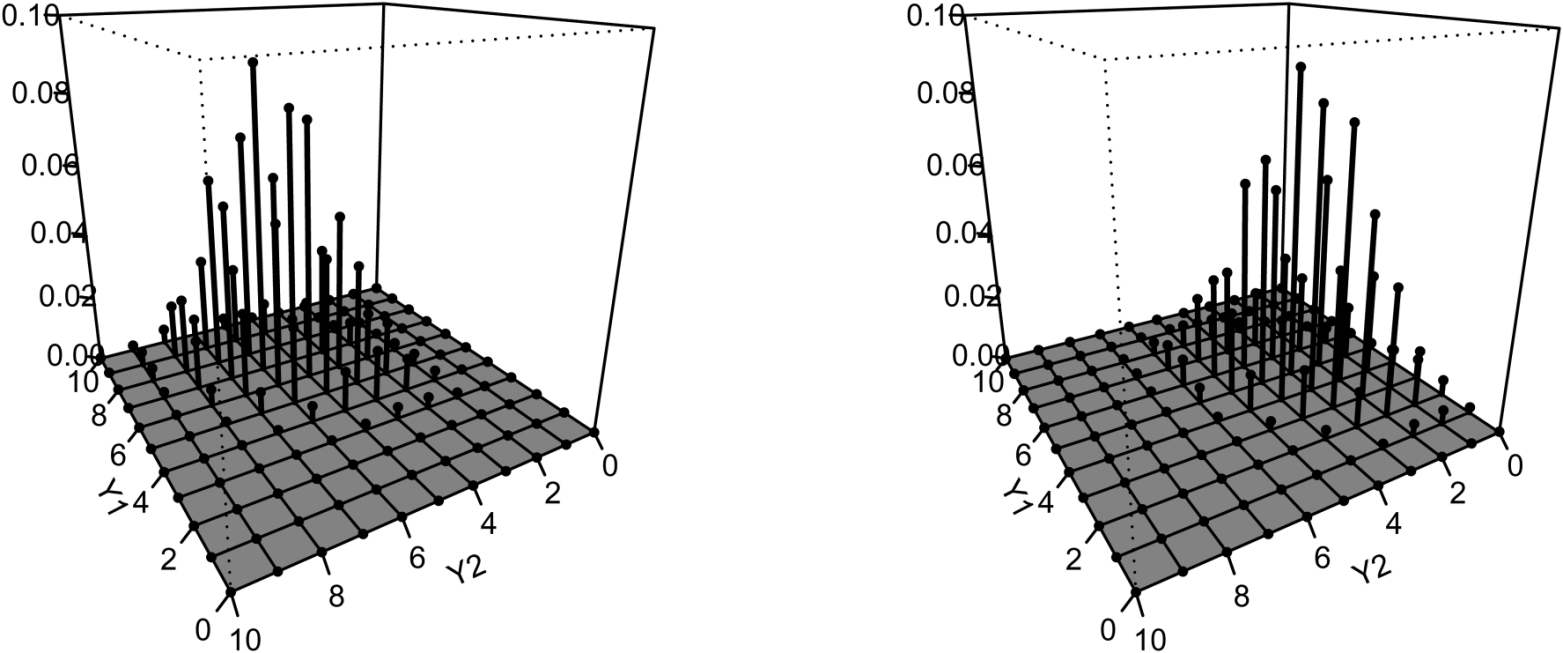
Fitted bivariate binomial distribution for immediate recall ( $Y_{1}$) and delayed recall ( $Y_{2}$). Left hand side: conditional on age 50; Right hand side: conditional on age 80.

We draw the three-dimensional plot Figure 4 of the bivariate binomial distribution based on the result in Table 2 with random effect ${\hat{b}}_{i}$s equal to the mean of estimated $b_{i}$ in (36). The left-hand side density plot is conditional on age 50, and the right-hand side density is conditional on age 80. It is clear that as age increases, the individual’s number of recalled words are likely to decrease. At the age of 50, the number of immediate-recall words is concentrated in 4–10. When the age reaches 80, the number of immediate-recall words is reduced to 2–8. This change is more obvious in the number of delayed-recall words. At the age of 50, the number of delayed-recall words is concentrated in 3–8. However, the number of delayed-recall words for most individuals drops sharply to 0–5 when individuals’ age reaches 80. Numerically, we can confirm this conclusion via the corresponding expectations: $E(Y_{1},Y_{2}|t=50)=(6.8,5.6)$; $E(Y_{1},Y_{2}|t=80)=(4.6,3.0)$.

**Figure 5.**
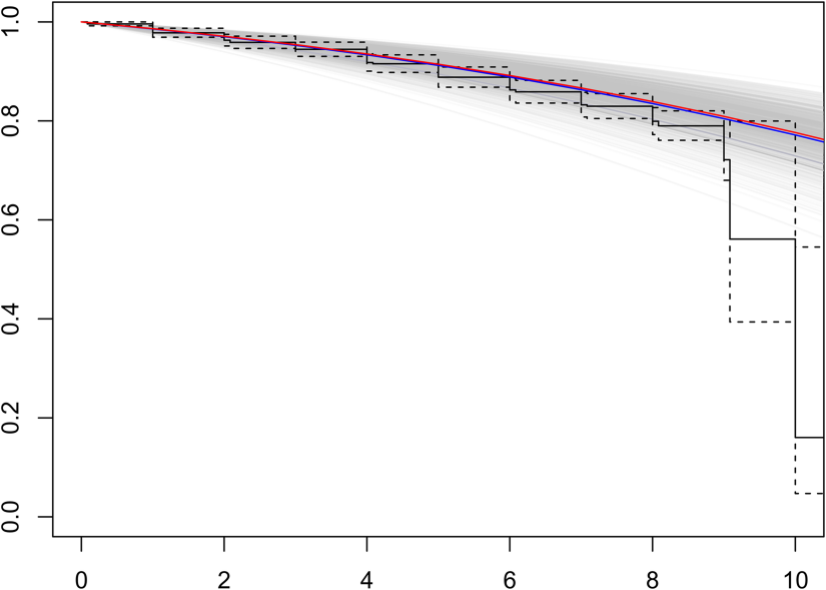
Comparison of K-M survival curves: predicted survival curves (grey lines), the mean of those survival curves (blue line) and the median of survival curves (red line).

Given the observed age and corresponding responses, we expect that our joint model can predict the risk of death. To prove this, we plot predicted survival curves based on the calculated random effects and compared these curves to the observed survival curve (i.e. the K-M curve). Figure 5 shows the comparison of Kaplan–Meier survival curves and predicted model survival curves based on Model *INT.G*2 with (15) j=1. The blue line shows the mean of predicted survival curves, and the red line shows the median of predicted survival curves. Only the baseline test score is used in Figure 5. The comparison plot illustrates that our joint model has a relatively better predictive ability for individual risk of death.

## 6 Conclusion

Joint models are constructed to analyze longitudinal and time-to-event data in ageing research. Since the tests used in the application are based on discrete values, and we are interested in two tests, the bivariate extension of the binomial distribution with mixed-effects regression is specified to model the longitudinal data. The bivariate extension of the binomial distribution allows modelling for correlation between the responses. The Gompertz hazard model and the Weibull hazard model are explored for the time-to-event data. The two models are joined together via the link function Δ, which contains the random effect b. The random effect is used to capture the dependence across time and the dependence between responses.

The joint model is used to analyze the ELSA data. Model comparison is undertaken by applying the Akaike information criterion. The AIC of the joint model with random intercept link function is smaller than the joint model using random slope link function, based on the same expression. When the link function equals to random slope for both responses, we got unexpected results: the slowly decline of the cognitive function over time is correlated with the high risk of death or dementia. The cause of this phenomenon needs further investigation.

We used the shared random-effects model to construct joint models in this paper. This model offers a flexible way to measure the correlation between the responses and the risk of event, i.e. the random effect follows a distribution instead of being equated to a fixed value. Instead of using random effects to capture correlation between responses, the joint model in this paper uses a separate parameter to model the correlation between responses. This allows us to more clearly identify the relationship between two responses.

Our modelling framework has some disadvantages. Firstly, it is computationally intensive since we need to calculate the double integral. Secondly, the distribution of random effects is set in advance. For example, we use the bivariate normal distribution in this paper. The multivariate normal distribution is the standard modelling choice for random effects in longitudinal submodels.^3^ Pantazis and Touloumi^45^ investigated the misspecification of the random effects distributions. They conclude that the fixed effects parameter estimates are fairly robust, except for the parameter estimates in the time-to-event sub-model. However, the SEs may be underestimated for severely skewed distribution. If the actual distribution is different from the normal distribution, it may lead to biased estimates.

There are still some aspects that can be used as starting points for further research. Firstly, we use linear predictors and logistic regression to model the probability in the bivariate binomial distribution. In further research, the linear effect of age in the logistic regression can be modelled more flexibly by using B-splines or other semi-parametric methods. Secondly, there are restrictions imposed by the shared random-effects model, which we have discussed earlier in this paper. Thirdly, in this paper, we construct a bivariate joint model whereas in longitudinal data there may be more than two responses. It is possible to construct the corresponding multivariate extended binomial distribution based on Altham and Hankin,^30^ and use this distribution in our joint model. However, this will imply a computational challenge with respect to integrating out the random effects. An alternative might be to use a Bayesian approach; for a recent development in this area see the *JMbayes* package.^25^ Lastly, as we mentioned in the ‘Models section, our framework is flexible in its choice of parametric hazard models, i.e. it is easy to replace the Gompertz hazard model and the Weibull hazard model by other parametric hazard models. Joint models using other parametric hazard models are worth being investigated.

To conclude, in this paper we construct a bivariate joint model to investigate the change in cognitive function. This bivariate joint model is general and can be used in a wide range of bivariate discrete-valued tests in ageing research. We discussed the shared part which is contained in both the longitudinal model and survival model. We hope our discussion will provide ideas for constructing other bivariate joint models in ageing research.
